# Watery Diarrhea Is Not Always Clostridioides difficile: A Case Report of Aeromonas hydrophila Gastroenteritis

**DOI:** 10.7759/cureus.51940

**Published:** 2024-01-09

**Authors:** Sahrudh Dharanendra, Aaron S Gillet, Bashir Geer, Mary Ann Kirkconnell Hall, Patricia R Hwang

**Affiliations:** 1 Department of Medicine, Emory University School of Medicine, Atlanta, USA; 2 Department of Medicine, Division of Hospital Medicine, Emory University School of Medicine, Atlanta, USA

**Keywords:** aeromonas infection, clostridioides difficile -associated diarrhea, acute watery diarrhea, gastroenteritis, aeromonas hydrophila

## Abstract

*Aeromonas* species can cause acute gastroenteritis but are much less commonly observed in the hospital setting than other bacteria. Most cases of *Aeromonas hydrophila* gastroenteritis reported in the literature have occurred in pediatric, elderly, and/or immunocompromised patients.

We present a case of subacute watery diarrhea due to *A. hydrophila *infection in an otherwise healthy 48-year-old female patient with prior abdominal surgeries and recent hospitalization for a catheter-associated urinary tract infection (CAUTI) for which she received antibiotics. The patient presented with 10 days of increasingly frequent non-bloody, watery, foul-smelling diarrhea as well as decreased oral intake, cramping bilateral upper abdominal pain, chills, and malaise.

Initial diagnoses considered included *Clostridioides difficile* in the setting of CAUTI and antibiotic use, small intestinal bacterial overgrowth, dumping syndrome related to bariatric surgery, and malabsorption. A computed tomography scan of her abdomen/pelvis, admission labs, and flexible sigmoidoscopy showed no relevant findings. Stool cultures eventually returned positive for *A. hydrophila.*

The case is an uncommon presentation of *Aeromonas* infection that could be easily missed while other diagnoses are pursued. Early treatment of *Aeromonas* infection can be crucial in preventing advanced forms of disease such as septicemia and necrotizing fasciitis.

## Introduction

*Aeromonas* species have been recognized as an emerging cause of acute gastroenteritis [1-3], but are much less commonly observed in the hospital setting than other bacteria, including *Clostridioides difficile*, *Salmonella *spp., and *Escherichia coli* [4]. *C. difficile i*s a common causative organism of watery diarrhea following antibiotic use; cases of diarrhea due to *C. difficile* have been increasing in frequency worldwide in the past decade, with community prevalence as high as 20% in some outbreaks [5,6]. Although a few cases of *Aeromonas hydrophila* gastroenteritis in healthy adults have been reported in the literature [7,8], most have been in pediatric [9-12], elderly [8,13-16], and/or immunocompromised patients [13,17]. In surveys of fecal samples from outbreaks of diarrheal illnesses across the globe, *A. hydrophila* is infrequently found, even in comparison to other *Aeromonas* spp. [18-21], though its prevalence may be increasing [22].

We present a case of subacute watery diarrhea due to *A. hydrophila* infection in an adult female patient with prior abdominal surgeries and recent hospitalization for a catheter-associated urinary tract infection (CAUTI) for which she received antibiotics (nitrofurantoin and trimethoprim/sulfamethoxazole) at an outside hospital. The case is an uncommon presentation of *Aeromonas* infection that could be easily misdiagnosed/treated as due to *C. difficile.* Early treatment of *Aeromonas* infection can be crucial in preventing advanced forms of disease such as septicemia and necrotizing fasciitis [23].

## Case presentation

A 48-year-old woman initially presented with 10 days of increasingly frequent non-bloody, watery diarrhea and decreased oral intake. She described the stools as foul-smelling and exacerbated by oral intake. Other symptoms included cramping bilateral upper abdominal pain, chills, and malaise.

Her medical history was significant for postural orthostatic tachycardia syndrome status post multiple ablations, Roux-en-Y gastric bypass in 2004, and multiple abdominal adhesion removal surgeries. She denied recent travel history or change in diet, but did note recent antibiotic use for a urinary tract infection that required hospitalization. Of note, she reported one syncopal episode while using the bathroom before coming to the hospital.

Upon admission, vital signs were normal. On physical examination, the patient had epigastric tenderness and hyperactive bowel sounds with no distension. On visual inspection of the patient’s abdomen, numerous scars were visible, consistent with her surgical history. The rest of her examination was unremarkable.

A detailed case timeline is presented in Table 1.

**Table 1:** Detailed case timeline.

Hospital day	Significant events
-14	The patient was hospitalized at an outside facility for a cardiac ablation and was diagnosed with a catheter-associated urinary tract infection following the placement of a Foley catheter. She received nitrofurantoin and trimethoprim/sulfamethoxazole
-10	The patient began experiencing non-bloody, watery diarrhea and decreased oral intake
1	The patient was admitted for diarrhea and syncopal episodes. A computed tomography scan of the abdomen/pelvis was performed. A stool sample was collected for C. difficile testing, cultures, and ova and parasite testing. No acute events were reported overnight. Maintenance IV fluids were started
2	The patient reported not having any bowel movements. Stool calprotectin, serum gastrin, thyroid-stimulating hormone, free thyroxine 4, and 24-hour stool collection for fat and quantity were ordered
3	The patient experienced four episodes of watery diarrhea. She expressed food aversion. The patient was started on Lactobacillus and loperamide
4	The patient experienced multiple bouts of diarrhea, notably shortly after any attempted feeding
5	Unchanged bowel movements. The patient endorsed headache and mild continued abdominal pain. She was placed on peripheral parenteral nutrition per the recommendations of the nutrition team. Urinalysis showed positive nitrites and a small amount of blood in the urine. The patient’s stool culture grew A. hydrophila. She was started on doxycycline 100 mg twice per day for one week
6	Ciprofloxacin 500 mg twice per day (BID) for seven days was started. Doxycycline was stopped, and the patient was started on dicyclomine 10 mg once per day for stomach cramping. Flexible sigmoidoscopy was performed without any abnormalities detected
7	The patient was discharged after improvement in symptoms on the following medications: ciprofloxacin 500 mg BID for seven days, dicyclomine 10 mg qd, and Saccharomyces boulardii 250 mg BID for 30 days. She was instructed to follow up with the outpatient gastroenterology service and stool cultures in two weeks

Diagnostic assessment

The initial non-exhaustive list of diagnoses considered included *C. difficile*, small intestinal bacterial overgrowth, dumping syndrome related to bariatric surgery, and malabsorption. *C. difficile* was considered in the setting of CAUTI and prior antibiotic use along with the patient’s presenting symptoms. Small intestine bacterial overgrowth, dumping syndrome, and malabsorption were considered in the setting of prolonged watery diarrhea in a patient with an extensive history of prior abdominal surgeries but were tentatively ruled out due to her symptomology; even after she was nil per os, she continued to have intermittent diarrhea, which suggested that the causes were not related to intake.

A computed tomography scan of her abdomen/pelvis showed fluid-filled loops of small bowel in the left lower quadrant without distention, but no acute findings. Her admission labs showed anion gap metabolic acidosis and no other relevant findings. *C. difficile* testing returned negative. She underwent a flexible sigmoidoscopy that showed no acute findings.

Later, her stool culture returned positive for *A. hydrophila*, which was included in the original culture request.

Therapeutic Intervention

The intervention included maintenance IV fluids for rehydration, ciprofloxacin/doxycycline for antibiotic action, *Lactobacillus*/*Saccharomyces boulardii* for probiotic activity, loperamide for diarrheal symptomatic treatment, and dicyclomine for anti-spasmodic activity for stomach cramping.

She was started on doxycycline, which was then changed to ciprofloxacin per the recommendation of the infectious disease team due to the patient’s variable resistance to doxycycline. She continued to receive loperamide and *Lactobacillus *with rapid improvement of her symptoms.

Outcome and follow-up

After seven days in the hospital, the patient was discharged on *S. boulardii*, dicyclomine, and ciprofloxacin. She was scheduled for and completed follow-up with outpatient gastroenterology physicians.

## Discussion

*A. hydrophila* is a gram-negative bacillus that is oxidase- and catalase-positive and frequently found in aquatic environments [24]. Although *A. hydrophila *gastroenteritis is a recognized foodborne illness that is more prevalent in the pediatric population, it should be considered in an atypical presentation of diarrhea in adults [9,11,25,26]. Gastroenteritis caused by this bacterium often presents in an acute or subacute manner with watery diarrhea that is treatable with medication but can often be mistaken for *C. difficile* [12]. Occasionally, patients with extensive surgical histories or hospital stays can develop *Aeromonas* infections leading to subacute diarrhea [27]. *A. hydrophilia *produces an enterotoxin through the *ACT *gene, which functions by binding cholesterol, and then aggregating the lipids to interact with mucosal cells of the gastrointestinal system and be internalized. Once internalized, they generate an inflammatory response and cause villi degeneration and mucoid-producing cells, leading to diarrhea (occasionally bloody) [28].

*Aeromonas *more commonly leads to infection of wounds, cellulitis, and the urinary tract, with gastroenteritis being an atypical presentation [7,17]. Although *Aeromonas* often presents as a urinary tract infection, our patient did not have clinical signs or symptoms of a urinary tract infection on admission. She did, however, report being treated for CAUTI several weeks before admission. It is possible that the *Aeromonas* infection began as a urinary tract infection that led to severe gastroenteritis.

Prior abdominal manipulations and gastrointestinal illness may also be factors for *A. hydrophila* infection. A report of surgical site infections in Spanish hospitals showed nine cases of *A. hydrophila* infection [29]. In all cases reviewed in the study, the patients were found to have preexisting gastrointestinal or biliary disease, suggesting a possible link between abdominal surgery and preexisting gastrointestinal conditions as risk factors for *Aeromonas* infections.

*A. hydrophila* represents a relatively uncommon pathogenic cause of severe diarrhea and should be considered in adult patients with risk factors such as a history of gastrointestinal surgery, gastrointestinal disease, and CAUTI if a patient tests negative for *C. difficile*. Our case presentation adds to the body of literature and further highlights that though *Aeromonas* diarrhea is much more common in the pediatric population, it can also present in the adult population. Our patient had no specific water-borne exposures and reported drinking only bottled water; her presentation was unusual in that it was more likely associated with her CAUTI/previous abdominal surgeries than with a water-associated exposure.

## Conclusions

In this case, *Aeromonas *infection presented in an otherwise healthy adult with watery diarrhea that could have been easily misdiagnosed or mistreated as resulting from another pathogen. Though infrequently observed in adults, *Aeromonas *infection is important to consider in the differential. Delayed diagnosis can lead to unnecessary antibiotic usage or complications from antibiotic selection, and untreated *Aeromonas* may progress to bacteremia/septicemia and necrotizing fasciitis.
